# *U2AF1* Mutations in Chinese Patients with Acute Myeloid Leukemia and Myelodysplastic Syndrome

**DOI:** 10.1371/journal.pone.0045760

**Published:** 2012-09-19

**Authors:** Jun Qian, Dong-ming Yao, Jiang Lin, Wei Qian, Cui-zhu Wang, Hai-yan Chai, Jing Yang, Yun Li, Zhao-qun Deng, Ji-chun Ma, Xing-xing Chen

**Affiliations:** 1 Department of Haematology, Affiliated People's Hospital of Jiangsu University, Zhenjiang, Jiangsu, People's Republic of China; 2 Laboratory Center, Affiliated People's Hospital of Jiangsu University, Zhenjiang, Jiangsu, People's Republic of China; 3 Department of Laboratory Medicine, Affiliated People's Hospital of Jiangsu University, Zhenjiang, Jiangsu, People's Republic of China; University of Barcelona, Spain

## Abstract

Somatic mutations of *U2AF1* gene have recently been identified in myelodysplastic syndrome (MDS) and acute myeloid leukemia (AML). In this study, we analyzed the frequency and clinical impact of *U2AF1* mutations in a cohort of 452 Chinese patients with myeloid neoplasms. Mutations in *U2AF1* were found in 2.5% (7/275) of AML and 6.3% (6/96) of MDS patients, but in none of 81 CML. All mutations were heterozygous missense mutations affecting codon S34 or Q157. There was no significant association of *U2AF1* mutation with blood parameters, FAB subtypes, karyotypes and other gene mutations in AML. The overall survival (OS) of AML patients with *U2AF1* mutation (median 3 months) was shorter than those without mutation (median 7 months) (*P* = 0.035). No difference in the OS was observed between MDS patients with and without *U2AF1* mutations. Our data show that *U2AF1* mutation is a recurrent event at a low frequency in AML and MDS.

## Introduction

Acute myeloid leukemia (AML) is characterized by autonomous proliferation and impaired differentiation of hematopoietic progenitors but is a genetically and phenotypically heterogeneous disease. The development of AML is associated with accumulation of acquired genetic alterations and epigenetic changes in hematopoietic progenitor cells that induces normal hematopoietic progenitor cell to loss the ability of self-renewal and differentiation to various mature cell lineages, to transform into a leukemic stem cell, and to accumulate in bone marrow [1], [2]. In recent years, an increasing number of gene mutations have been identified involved in the pathogenesis of the disease and have been shown to be correlated with prognosis of AML patients [3]. Some gene mutations have been further introduced into the current World Health Organization (WHO) classification [4].

U2AF1 (U2AF35), an essential component of the U2 small nuclear ribonucleoprotein auxiliary factor (U2AF), plays an important role in the splicing process in which functional mRNA is generated from pre-mRNA [5], [6]. The disruption of interactions of several factors involved in the splicing process can cause various types of mutations in an ever increasing number of genes [7]. Recently, somatic mutations in *U2AF1* were discovered in myelodysplastic syndrome (MDS) and mainly occurred in two codons (Ser34 and Q157) [8], [9]. Patients with *U2AF1* mutations had an increased probability of progression from MDS to AML, however, they had similar overall survival as those with wild-type *U2AF1* [8]. The prognostic impact and clinical characteristics of patients with *U2AF1* mutations in AML are unknown. Thus, in this study we investigated the frequency and prognostic influence of *U2AF1* mutations in a cohort of patients with AML, MDS and chronic myeloid leukemia (CML) patients.

## Materials and Methods

### Patients' samples

This study was approved by the Ethics Committee Board of Affiliated People's Hospital of Jiangsu University. Bone marrow aspirates or peripheral blood samples of patients with various hematologic malignancies were collected after informed consent written. The patients included 275 primary AML, 96 primary MDS, 81 CML (61 at chronic phase, 4 at accelerated phase, 16 at blast crisis). These hematological malignancies were diagnosed according to the French-American-British Cooperative Group Criteria and the 2008 World Health Organization proposal [4], [10]. Karyotypes were classified according to reported previously [11], [12]. Bone marrow specimens obtained at the time of complete hematologic remission from three patients (one AML, one RAEB-1 and one RAEB-2) with *U2AF1* mutations at initial diagnosis and peripheral blood from 103 healthy individuals were used as control. The mononuclear cells were separated by density-gradient centrifugation using Ficoll. Subsequently, genomic DNAs were extracted using the Genomic DNA Purification Kit (Gentra, USA) according to the manufacturer's instructions.

**Table 1:** The clinical and hematopoietic parameters of 13 patients with *U2AF1* mutations.

ID	Sex/Age (years)	Diagnosis	WBC (×10^9^/L)	Hemoglobin (g/L)	Platelet (×10^9^/L)	Karyotype	Survival time (months)	*U2AF1* mutation
1	M/20	AML-M2	130.2	107	69	+8	3	S34Y
2	M/76	AML-M1	97.0	40	33	No data	1	Q157P
3	F/29	AML-M2	75.1	76	50	+8	1	S34Y
4	M/44	AML-M4	3.9	64	48	−7	9	Q157R
5	M/60	AML-M2	3.5	54	30	N	6	S34F
6	F/80	AML-M2	0.9	56	31	N	5	S34Y
7	M/66	AML-M5	37.2	65	42	1p+,−5,22q+,+mar1,+mar2	No data	S34Y
8	M/86	RAEB-1	2.3	43	31	No data	4+	Q157P
9	M/31	RAEB-2	1.4	51	30	N	4+	S34Y
10	F/31	RCMD-RS	2.6	79	101	del(5)(q21q34)	45	S34F
11	M/28	RAEB-2	2.4	56	44	N	40	S34F
12	M/67	RAEB-1	1.6	66	130	N	22	S34F
13	F/40	RCMD-RS	3.0	41	88	+8	1+	S34Y

M, male; F, female; N, normal;.

### Mutation scanning

Primers to amplify the coding sequences of *U2AF1* (GenBank NC_000021.8) are listed in Table S1. genomic DNA samples were amplified in a final volume of 25 μl containing 1× PCR buffer (Invitrogen, Merelbeke, Belgium), 0.2 mmol/L of each dNTP, 2.5 mmol/L of MgCl_2_, 0.4 μmol/L of both forward and reverse primers, 0.8 μmol/L of oligonucleotide calibrators [13], 1× LCgreen Plus (Idaho Technology Inc. Salt Lake City, Utah), and 1 U Taq polymerase (MBI Fermentas, Canada). All PCR amplicons were generated on a 7300 Thermo cycler (Applied Biosystems, Foster City, CA, USA) using the PCR program as follows: an initial denaturation step was started at 95°C for 5 minutes, followed by 40 cycles of denaturation at 94°C for 30 seconds, annealing at melting temperature for 30 seconds, and an extension at 72°C for 30 seconds. Mutation scanning was performed for PCR products using high-resolution melting analysis (HRMA) with the LightScanner^TM^ platform (Idaho Technology Inc. Salt Lake City, Utah) according to the protocol reported previously [14].

**Table 2:** Distribution of *U2AF1* mutations in AML and MDS.

	*U2AF1* mutation	Wild-type	*P*
AML	7	268	
Sex, male/female	5/2	149/119	0.471
Median age at diagnosis, years (range)	60 (20–80)	47 (15–93)	0.402
Median WBC at diagnosis, ×10^9^/L (range)	37.2 (0.9–130.2)	14 (0.5–528)	0.690
Median hemoglobin at diagnosis, g/L (range)	64 (40–107)	74 (32–147)	0.196
Median platelets at diagnosis, ×10^9^/L (range)	42 (30–69)	38 (3–447)	0.634
FAB, no.			0.770
M0	0	1	
M1	1	33	
M2	4	106	
M3	0	42	
M4	1	42	
M5	1	33	
M6	0	11	
Karyotype classification			0.152
Favorable	0	72	
Intermediate	4	145	
Poor	2	30	
No data	1	21	
Gene mutations			
C-KIT (+/−)	0/7	13/255	1.000
NPM1 (+/−)	2/5	20/248	0.100
FLT3-ITD (+/−)	0/7	14/254	1.000
IDH1/IDH2 (+/−)	0/7	12/256	1.000
DNMT3A (+/−)	0/7	14/254	1.000
MDS	6	90	
Sex, male/female	4/2	54/36	1.000
Median age at diagnosis, years (range)	36 (28–86)	60 (20–85)	0.134
Median WBC at diagnosis, ×10^9^/L (range)	2.4 (1.4–3.0)	2.8 (0.6–82.4)	0.129
Median hemoglobin at diagnosis, g/L (range)	54 (41–79)	62 (26–128)	0.237
Median platelets at diagnosis, ×10^9^/L (range)	66 (30–130)	60 (1–1176)	0.745
WHO, no.			0.571
5q-	0	3	
RA/RARS/RT	0	11	
RCMD/RCMD-RS	2	41	
RAEB-1	2	18	
RAEB-2	2	17	
Karyotype classification			0.386
Favorable	4	64	
Intermidiate	1	16	
Poor	0	8	
No data	1	2	
IPSS			0.449
Low	0	9	
Int-1	4	56	
Int-2	0	17	
High	0	6	
No data	0	2	
Gene mutations			
IDH1/IDH2 (+/−)	0/6	5/85	
DNMT3A (+/−)	0/6	4/86	

WBC indicates white blood cell count at diagnosis; IPSS, International Prognostic Scoring System; WHO, World Health Organization; FAB, French-American-British classification; RA, refractory anemia; RARS, refractory anemia with ringed sideroblasts; RT, refractory thrombocytopenia; RCMD, refractory cytopenia with multilineage dysplasia; RCMD-RS, refractory cytopenia with multilineage dysplasia with ringed sideroblasts; RAEB, refractory anemia with excess of blasts;.

*FLT3* internal tandem duplication (ITD), *NPM1*, *IDH1*/*IDH2* and *DNMT3A* mutations were detected as reported previously [15]–[18]. *C-KIT* mutation was also detected using PCR-HRMA.

### DNA sequencing

To confirm HRMA results, DNA sequencing was also performed in all samples identified by HRMA. PCR products were directly sequenced on both strands using an ABI 3730 automatic sequencer.

### Statistics

Statistical analysis was performed using the SPSS 13.0 software package (SPSS, Chicago, IL, USA). Pearson Chi-square analysis and Fisher exact test were carried out to compare the difference of categorical variables between patient groups. Mann-Whitney's U-test was carried out to compare the difference of continuous variables between patient groups. Survival was analyzed according to the Kaplan-Meier method. For all analyses, a *P*-value of less than 0.05 (two-tailed) was considered statistically significant.

## Results and Discussion

HRMA could easily distinguish *U2AF1* mutations (S34Y and Q157R) with the sensitivity of 5% in a background of wild-type DNA, higher than that obtained by direct DNA sequencing (10%) (Figures S1, S2, S3 and S4).

In the cohort of 452 patients with myeloid malignancies, a heterozygous *U2AF1* mutant was found to in 13 cases with AML or MDS, but in none with CML. The representative results of HRMA and directing sequencing of *U2AF1* mutations in AML and MDS were presented in Figures S5, S6, S7 and S8. The clinical characteristics of all patients with *U2AF1* mutations were shown in Table 1. *U2AF1* mutation, positive in the bone marrow samples from three individuals (case 4,11 and 12, Table 1) at initial diagnosis, disappeared after the first complete remission. Furthermore, *U2AF1* mutation was not present in all healthy controls. These results support the somatic nature of *U2AF1* mutations.

Heterozygous *U2AF1* mutations were found in 7 (2.5%) of 275 AML patients (Table 1), including 3 S34Y, 2 S34F and 2 Q157 (1 Q157P and 1 Q157R) mutations. There were no difference in sex, age, blood parameters, FAB subtypes, and karyotype classification between cases with and without mutations (*P*>0.05, Table 2). Makishima et al reported higher occurrence (9.1%, 5/55) of *U2AF1* mutations in primary AML [19]. A larger cohort of Yoshida et al revealed 2% (3/151) of *U2AF1* mutations in Japanese AML population [9]. *U2AF1* mutations mainly occurred in the FAB subtypes of M1, M2, M4 and M5 [9], [19]. Interestingly, all five *U2AF1* mutations found by Makishima et al occurred in cytogenetically abnormal AML, including two cases with monosomy 7 [19]. However, two cases with *U2AF1* mutations were identified with trisomy 8 besides one with monosomy 7 in our AML group. More patients should be investigated to determine the association of *U2AF1* mutations with karyotypes. *C-KIT*, *FLT3-ITD*, *NPM1*, *IDH1*/*IDH2* and *DNMT3A* mutations were also detected. Among the patients with *U2AF1* mutations, only two cases had *NPM1* mutation. There was no significant association of *U2AF1* mutation with other molecular alterations (Table 2).

Follow-up data were obtained for 150 AML patients. There was no significant difference in complete remission rate between patients with and without *U2AF1* mutation (57.1% vs 72.9%, *P* = 0.398). The median follow-up duration of the patients was 7 months (range, 1–73 months). M3 subtype was excluded from survival analysis due to different therapy and prognosis. The estimated 50% survival time of the remaining 126 patients was 7 months. The overall survival (OS) of AML patients with *U2AF1* mutation (median 3 months, 95% confidence interval 0–7.8 months) was shorter than those without mutation (median 7 months, 95% confidence interval 4.8–9.2 months) (*P* = 0.035, Figure S9). However, there was no difference in disease-free survival between the patients with and without *U2AF1* mutation. Two patients with *U2AF1* mutation died early after initial diagnosis due to central nervous system involvement and sepsis respectively. Furthermore, a multivariate analysis for outcomes could not be performed because of the small sample size of patients with mutations. A larger cohort from a clinical trial will be needed to definitively address the effect of *U2AF1* mutations on outcomes.

6 (6.3%) MDS cases were identified with heterozygous *U2AF1* mutations (3 S34F, 2 S34Y, and 1Q157P) (Table 1). No significance in sex, age, blood parameters, WHO subtypes, and IPSS classification was observed between MDS patients with and without *U2AF1* mutations (*P*>0.05, Table 2). 7.3%–8.8% of *U2AF1* mutations have been reported in primary MDS recently [8], [9], [19]–[21]. All 72 *U2AF1* mutations in MDS including ours, which could be almost found in each FAB or WHO subtype, exclusively occurred at the highly conserved sites of exon S34 and Q157 with a rare exception of A26V, E159, or R156H mutation in three subjects [9], [17], [18]. Significant association of *U2AF1* mutations has not been identified with specific karyotypes. Although Damm et al [21] found that the association of del20q with *U2AF1* mutation, no *U2AF1* mutation was observed in all four cases with del20q in our group. Survival analysis was performed in 76 MDS cases with follow-up information. No difference in OS was observed between patients with and without *U2AF1* mutations (*P* = 0.821, Figure S9). The impact of *U2AF1* mutations on clinical outcome has been controversial in MDS [8], [19], [20]. Although Makishima et al considered *U2AF1* mutation as a factor predictive for shorter survival [19], other three studies did not find the association of *U2AF1* mutation with prognosis [8], [20], [21]. More cases with *U2AF1* mutations should be further studied to determine its prognostic relevance.

The definite role of *U2AF1* in the cancer pathogenesis has not been known. *U2AF1* mutation induces abnormal global RNA splicing which has been described in a wide variety of cancers [22]–[24]. Reduced expression of *U2AF1* was found in pancreatic cancer cells and correlated with mis-splicing of the cholecystokinin-B/gastrin receptor mRNA [25]. Additionally, knockdown of *U2AF1* reduces cell proliferation, induces G2/M arrest, and enhances apoptosis [26]. Moreover, a recent study demonstrated that S34F mutant had the same effect as *U2AF1* downregulation [9]. These results suggest that *U2AF1* mutant leads to loss of function and contributes to ineffective hematopoiesis and the cytopenias seen in MDS. Furthermore, subjects with *U2AF1* mutations were not restricted to a particular WHO subtypes, which indicates *U2AF1* mutation should be an early, initial genetic event in MDS.

In summary, mutations in *U2AF1* occur in patients with AML at a low frequency and are associated with a negative prognosis in AML which will require confirmation in a larger cohort.

## Supporting Information

Figure S1 **Results of a dilution series of S34Y** ***U2AF1*** **mutant in a background of wild-type DNA detected by HRMA.** 1: 0%, 1% and 2% mutant; 2: 5% mutant; 3: 10% mutant; 4: 25% mutant; 5: 50% mutant; 6: 100% mutant. A: normalized melting peaks; B: normalized difference curves. Mutated S34Y *U2AF1* was identified by HRMA at the maximal sensitivity of 5%. Although the shapes were similar, homozygous mutants could be distinguished from wild-type amplicons by Tm shift.
(DOC)

Figure S2 **Results of a dilution series of S34Y** ***U2AF1*** **mutant in a background of wild-type DNA detected by DNA sequencing.** The maximal sensitivity of 10% was obtained. Arrow showed the mutation site.
(DOC)

Figure S3 **Results of a dilution series of Q157R** ***U2AF1*** **mutant in a background of wild-type DNA detected by HRMA.** 1: 0%, 1% and 2% mutant; 2: 5% mutant; 3: 10% mutant; 4: 25% mutant; 5: 50% mutant; 6: 100% mutant. A: normalized melting peaks; B: normalized difference curves.
(DOC)

Figure S4 **Results of a dilution series of Q157R** ***U2AF1*** **mutant in a background of wild-type DNA detected by DNA sequencing.** The maximal sensitivity of 10% was obtained. Arrow showed the mutation site.
(DOC)

Figure S5 **HRMA screening of S34** ***U2AF1*** **mutations in MDS patients.** Grey lines represent wild-type S34 *U2AF1*; Blue line represents heterozygous S34F mutant in one MDS case; Orange line represents heterozygous S34Y mutant in one MDS case.
(DOC)

Figure S6 **Sequencing results of S34** ***U2AF1*** **mutations in AML and MDS patients.** A: heterozygous S34Y mutation (TCT→TAT); B: heterozygous S34F mutation (TCT→TTT). Arrow denotes mutation site.
(DOC)

Figure S7 **HRMA screening of Q157** ***U2AF1*** **mutations in AML patients.** Grey lines represent wild-type Q157 *U2AF1*; Red lines represent heterozygous Q157P mutant in one AML case.
(DOC)

Figure S8 **Sequencing results of Q157** ***U2AF1*** **mutations.** A: heterozygous Q157P mutation (CAG→CCG) in one case with AML-M1; B: heterozygous Q157R mutation (CAG→CGG) in one case with AML-M4; Arrow denotes mutation site.
(DOC)

Figure S9 **Overall survival of AML or MDS patients divided according to U2AF1 mutation status at diagnosis.** A: AML; B: MDS.
(DOC)

Table S1 **The sequences of primers used in PCR for HRMA or direct sequencing.**
(DOC)
