# Supplementary material for: U2AF1 Mutations in Chinese Patients with Acute Myeloid Leukemia and Myelodysplastic Syndrome
Source: PLoS One. 2012 Sep 19;7(9):e45760. doi: 10.1371/journal.pone.0045760 (PMC3446943; doi:10.1371/journal.pone.0045760)
Supplement: Figure S1 — Results of a dilution series of S34Y U2AF1 mutant in a background of wild-type DNA detected by HRMA. 1: 0%, 1% and 2% mutant; 2: 5% mutant; 3: 10% mutant; 4: 25% mutant; 5: 50% mutant; 6: 100% mutant. A: normalized melting peaks; B: normalized difference curves. Mutated S34Y U2AF1 was identified by HRMA at the maximal sensitivity of 5%. Although the shapes were similar, homozygous mutants could be distinguished from wild-type amplicons by Tm shift. (DOC) [file pone.0045760.s001.doc]

**Figure S1: Results of a dilution series of S34Y *U2AF1* mutant in a background of wild-type DNA detected by HRMA.** 1: 0%, 1% and 2% mutant; 2: 5% mutant; 3: 10% mutant; 4: 25% mutant; 5: 50% mutant ; 6 : 100% mutant. A: normalized melting peaks; B: normalized difference curves. Mutated S34Y *U2AF1* was identified by HRMA at the maximal sensitivity of 5%. Although the shapes were similar, homozygous mutants could be distinguished from wild-type amplicons by Tm shift.

**A
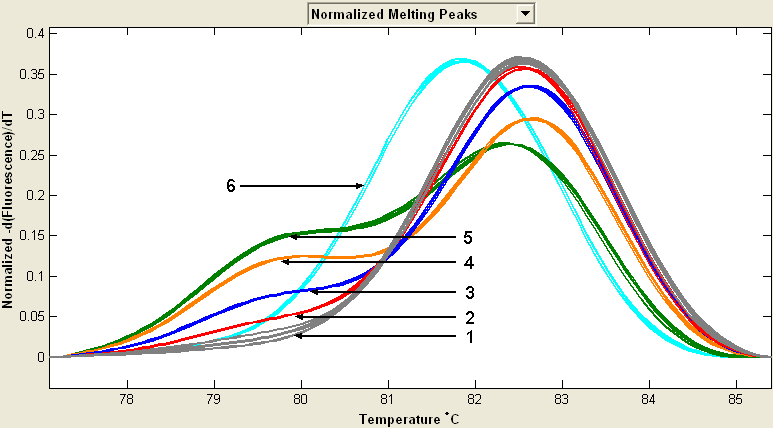
**

**B
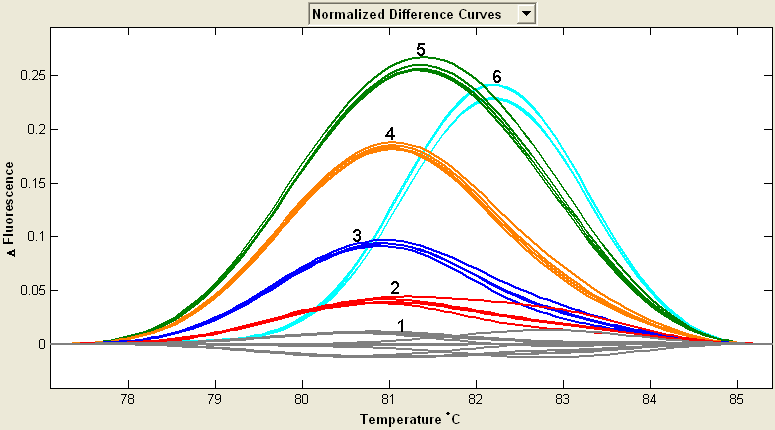
**
