# Supplementary material for: U2AF1 Mutations in Chinese Patients with Acute Myeloid Leukemia and Myelodysplastic Syndrome
Source: PLoS One. 2012 Sep 19;7(9):e45760. doi: 10.1371/journal.pone.0045760 (PMC3446943; doi:10.1371/journal.pone.0045760)
Supplement: Figure S2 — Results of a dilution series of S34Y U2AF1 mutant in a background of wild-type DNA detected by DNA sequencing. The maximal sensitivity of 10% was obtained. Arrow showed the mutation site. (DOC) [file pone.0045760.s002.doc]

**Figure S2: Results of a dilution series of S34Y *U2AF1* mutant in a background of wild-type DNA detected by DNA sequencing.** The maximal sensitivity of 10% was obtained. Arrow showed the mutation site.

**
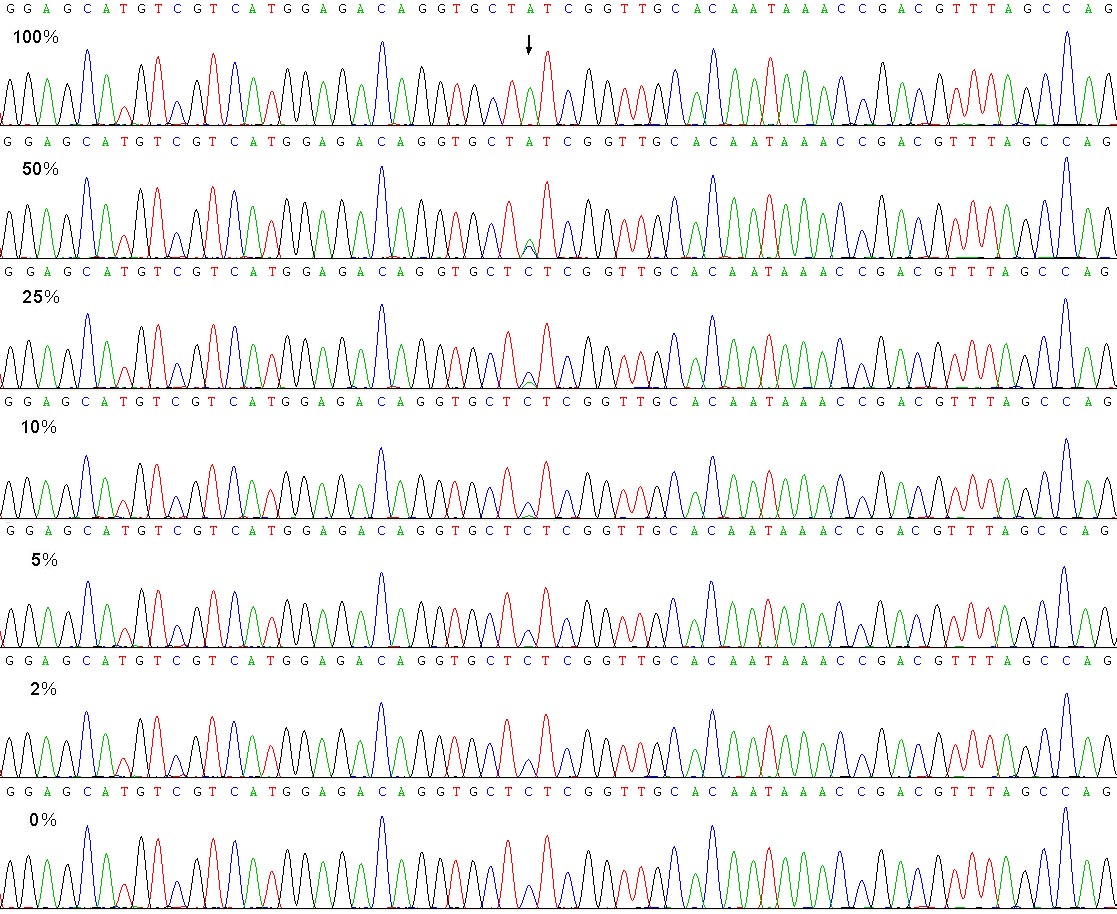
**
