# Supplementary material for: U2AF1 Mutations in Chinese Patients with Acute Myeloid Leukemia and Myelodysplastic Syndrome
Source: PLoS One. 2012 Sep 19;7(9):e45760. doi: 10.1371/journal.pone.0045760 (PMC3446943; doi:10.1371/journal.pone.0045760)
Supplement: Figure S5 — HRMA screening of S34 U2AF1 mutations in MDS patients. Grey lines represent wild-type S34 U2AF1; Blue line represents heterozygous S34F mutant in one MDS case; Orange line represents heterozygous S34Y mutant in one MDS case. (DOC) [file pone.0045760.s005.doc]

**Figure S5: HRMA screening of S34 *U2AF1* mutations in MDS patients.** Grey lines represent wild-type S34 *U2AF1*; Blue line represents heterozygous S34F mutant in one MDS case; Orange line represents heterozygous S34Y mutant in one MDS case.

**
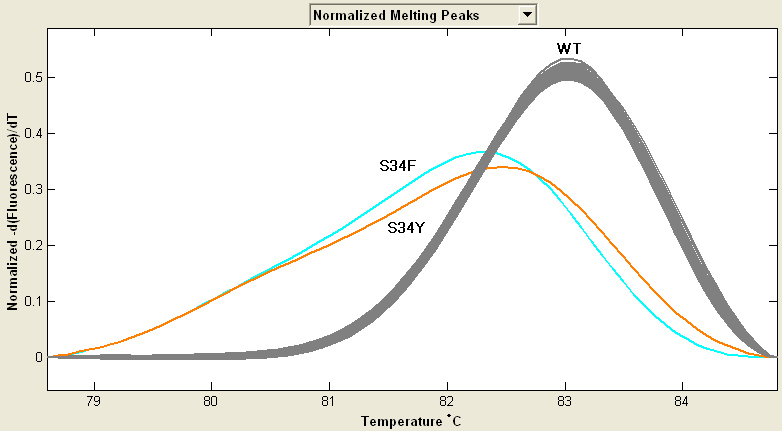
**
