# Supplementary material for: U2AF1 Mutations in Chinese Patients with Acute Myeloid Leukemia and Myelodysplastic Syndrome
Source: PLoS One. 2012 Sep 19;7(9):e45760. doi: 10.1371/journal.pone.0045760 (PMC3446943; doi:10.1371/journal.pone.0045760)
Supplement: Figure S6 — Sequencing results of S34 U2AF1 mutations in AML and MDS patients. A: heterozygous S34Y mutation (TCT→TAT); B: heterozygous S34F mutation (TCT→TTT). Arrow denotes mutation site. (DOC) [file pone.0045760.s006.doc]

**Figure S6: Sequencing results of S34 *U2AF1* mutations in AML and MDS patients.** A: heterozygous S34Y mutation (TCT→TAT); B: heterozygous S34F mutation (TCT→TTT). Arrow denotes mutation site.


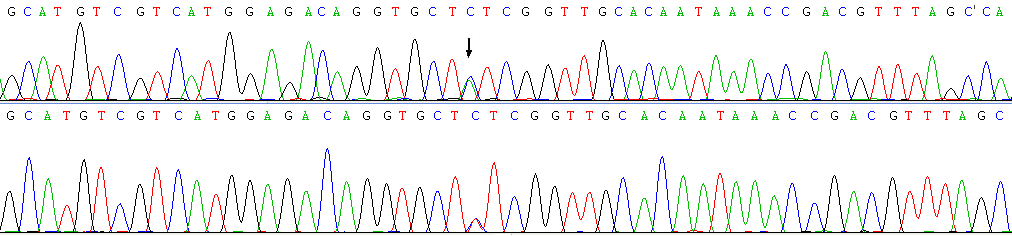


A

B
