# Supplementary material for: U2AF1 Mutations in Chinese Patients with Acute Myeloid Leukemia and Myelodysplastic Syndrome
Source: PLoS One. 2012 Sep 19;7(9):e45760. doi: 10.1371/journal.pone.0045760 (PMC3446943; doi:10.1371/journal.pone.0045760)

**Figure S7: HRMA screening of Q157 *U2AF1* mutations in AML patients.** Grey lines represent wild-type Q157 *U2AF1*; Red lines represent heterozygous Q157P mutant in one AML case.


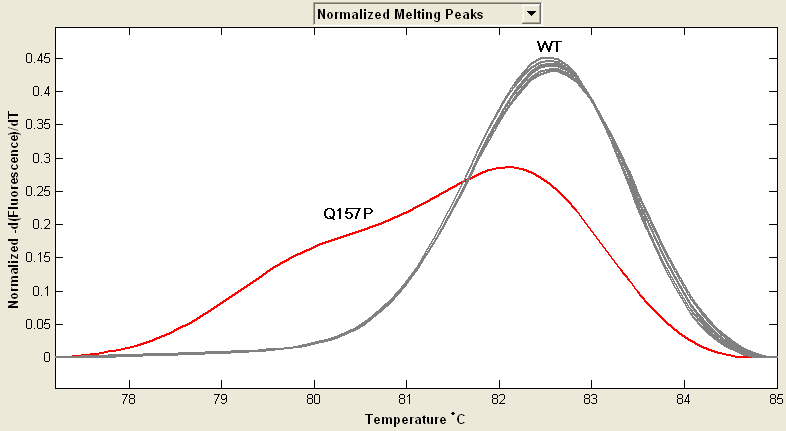

Supplement: Figure S7 — HRMA screening of Q157 U2AF1 mutations in AML patients. Grey lines represent wild-type Q157 U2AF1; Red lines represent heterozygous Q157P mutant in one AML case. (DOC) [file pone.0045760.s007.doc]
