# Supplementary material for: U2AF1 Mutations in Chinese Patients with Acute Myeloid Leukemia and Myelodysplastic Syndrome
Source: PLoS One. 2012 Sep 19;7(9):e45760. doi: 10.1371/journal.pone.0045760 (PMC3446943; doi:10.1371/journal.pone.0045760)
Supplement: Figure S8 — Sequencing results of Q157 U2AF1 mutations. A: heterozygous Q157P mutation (CAG→CCG) in one case with AML-M1; B: heterozygous Q157R mutation (CAG→CGG) in one case with AML-M4; Arrow denotes mutation site. (DOC) [file pone.0045760.s008.doc]

**Figure S8: Sequencing results of Q157 *U2AF1* mutations.** A: heterozygous Q157P mutation (CAG→CCG) in one case with AML-M1; B: heterozygous Q157R mutation (CAG→CGG) in one case with AML-M4; Arrow denotes mutation site.


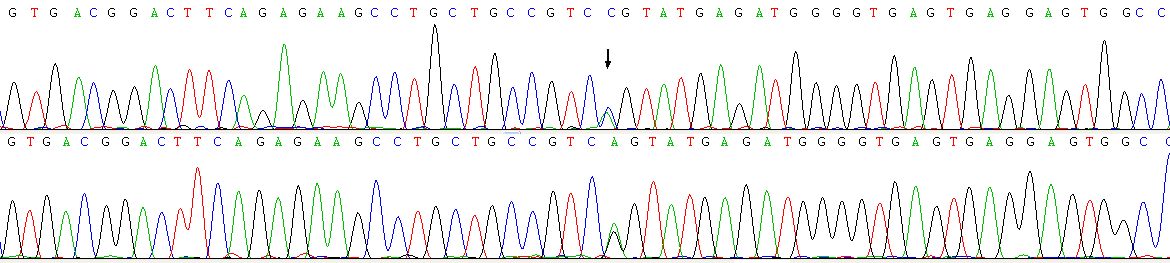


A

B
