# Supplementary material for: U2AF1 Mutations in Chinese Patients with Acute Myeloid Leukemia and Myelodysplastic Syndrome
Source: PLoS One. 2012 Sep 19;7(9):e45760. doi: 10.1371/journal.pone.0045760 (PMC3446943; doi:10.1371/journal.pone.0045760)

**Figure S9: Overall survival of AML or MDS patients divided according to U2AF1 mutation status at diagnosis.** A: AML; B: MDS.

A **
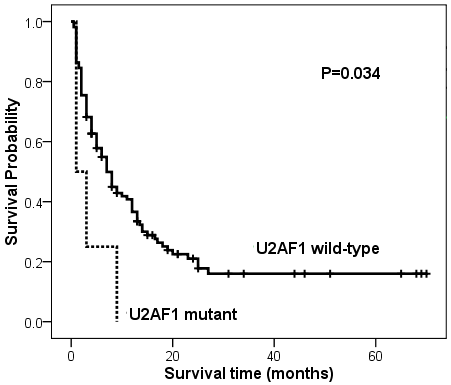
**

B
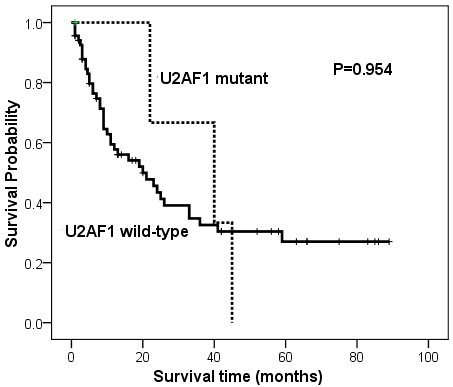

Supplement: Figure S9 — Overall survival of AML or MDS patients divided according to U2AF1 mutation status at diagnosis. A: AML; B: MDS. (DOC) [file pone.0045760.s009.doc]
