# Supplementary material for: U2AF1 Mutations in Chinese Patients with Acute Myeloid Leukemia and Myelodysplastic Syndrome
Source: PLoS One. 2012 Sep 19;7(9):e45760. doi: 10.1371/journal.pone.0045760 (PMC3446943; doi:10.1371/journal.pone.0045760)
Supplement: Table S1 — The sequences of primers used in PCR for HRMA or direct sequencing. (DOC) [file pone.0045760.s010.doc]

**Table S1: The sequences of primers used in PCR for HRMA or direct sequencing.**

| Exon | Forward (5’→3’) | Reverse (5’→3’) | Product  (bp) | Annealing temperature ( ºC) |
| --- | --- | --- | --- | --- |
| 1 | GGGTGACGTCTCCCGAG | TCCCACCGCCTCAACCA | 189 | 61 |
| 2 | AATAATCAGCTCTCATTTTCCCT | ATGTAGAAATTAACTGTCTTTGAAAAGAAC | 187 | 56 |
| 3 | TTAATTTTCCATAATATAAAGTTGTTGCGT | CGGGGAAGGAACTTGTATGA | 135 | 57 |
| 4 | ACCAGCAAGATTTCTGTTGT | AGAAGATCAACAGGTCTATCAGTCA | 120 | 57 |
| 5 | ACACTTTGTAATGTATAACGAGTCTT | TTGGCCCTGGCACACTAA | 199 | 57 |
| 6 | GATGGCAAGCACTTCTGTT | GTCACTGGCCACTCCTC | 189 | 58 |
| 7 | GCACACGAGGTAGTAAATGTC | GCCACTGAGCTTAACAACG | 170 | 58 |
| 8 | GTTTGGATGGCACGGTTT | ACACTTTCTAGCAGACATAAGGTA | 225 | 57 |
| 8 | GCGATTCTGAGCCATGC | ACTTCCACAAGAAATAAGTTACACC | 186 | 58 |
